# Supplementary figures and images for: Equus β-Defensin-1 Regulates Innate IMMUNE Response in S. aureus-Infected Mouse Monocyte Macrophage
Source: Animals (Basel). 2022 Oct 27;12(21):2958. doi: 10.3390/ani12212958 (PMC9654823; doi:10.3390/ani12212958)

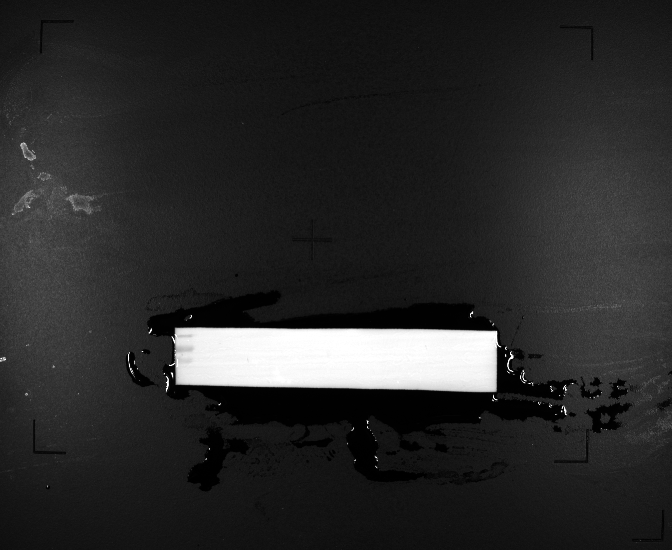

Supplement: Supplementary file 1 [file animals-12-02958-s001.zip › Western blot Original Images/AKT/001-bright.tif]

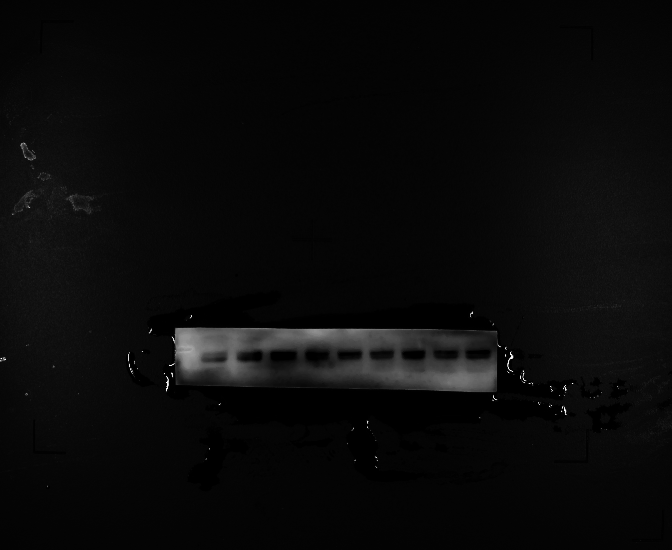

Supplement: Supplementary file 1 [file animals-12-02958-s001.zip › Western blot Original Images/AKT/001-merger.tif]

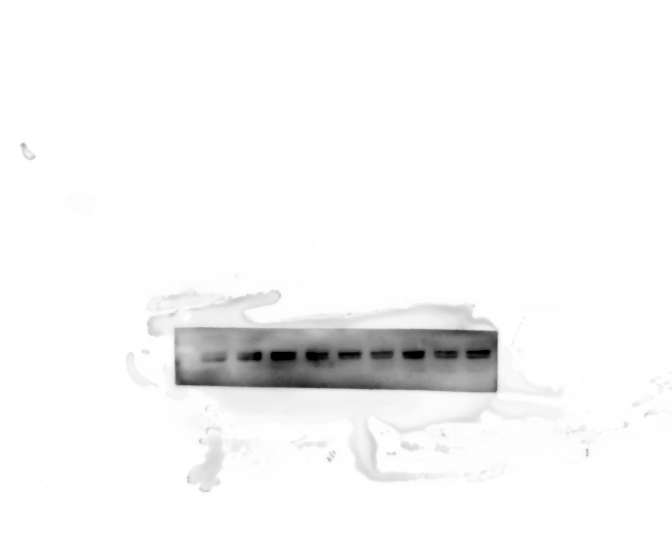

Supplement: Supplementary file 1 [file animals-12-02958-s001.zip › Western blot Original Images/AKT/001-shine.tif]

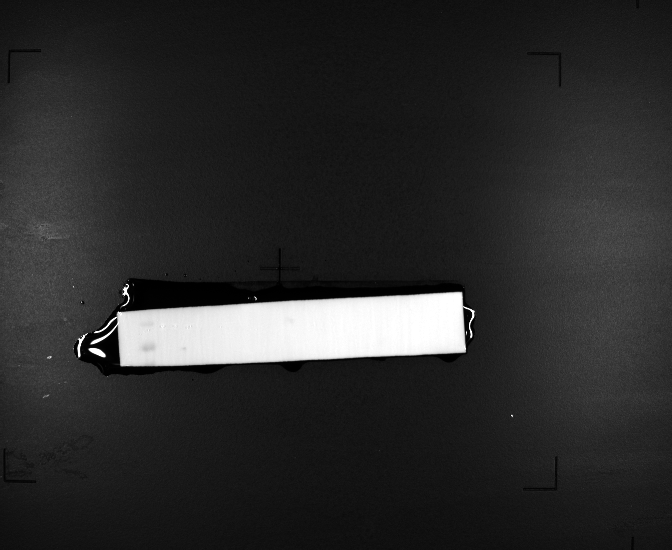

Supplement: Supplementary file 1 [file animals-12-02958-s001.zip › Western blot Original Images/GAPDH/001-bright.tif]

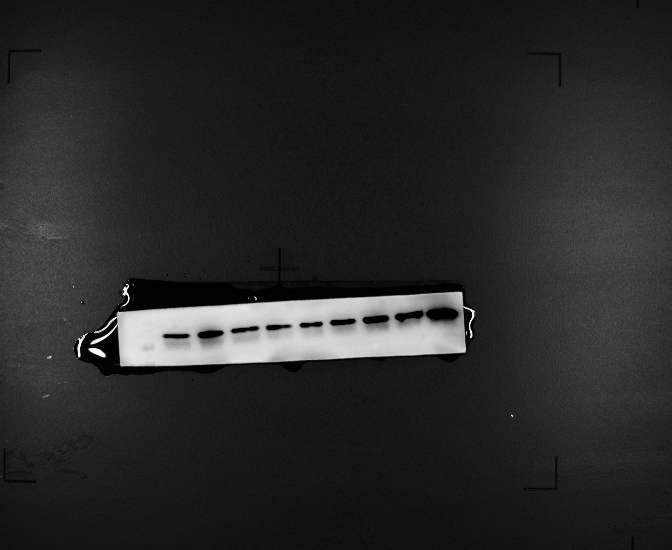

Supplement: Supplementary file 1 [file animals-12-02958-s001.zip › Western blot Original Images/GAPDH/001-merger.tif]

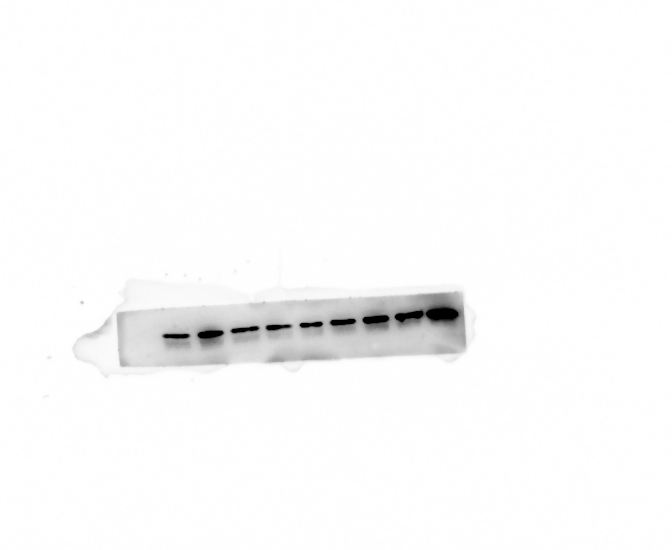

Supplement: Supplementary file 1 [file animals-12-02958-s001.zip › Western blot Original Images/GAPDH/001-shine.tif]

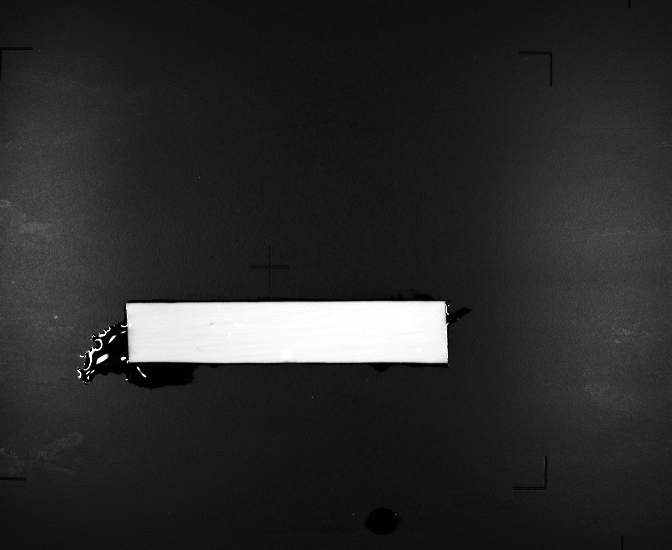

Supplement: Supplementary file 1 [file animals-12-02958-s001.zip › Western blot Original Images/IkB-a/001-bright.tif]

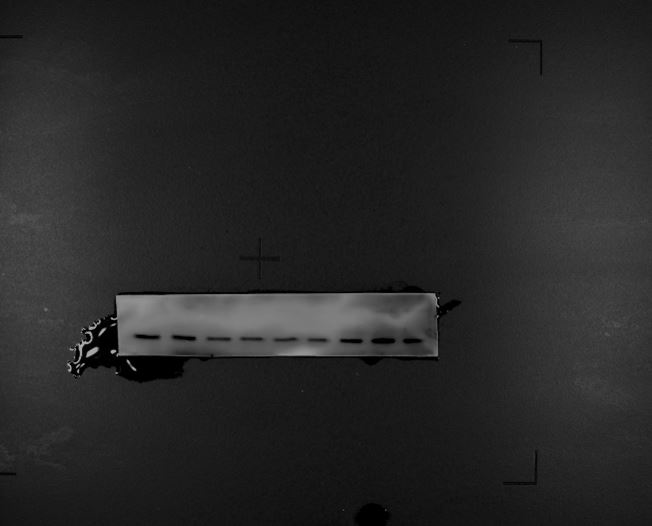

Supplement: Supplementary file 1 [file animals-12-02958-s001.zip › Western blot Original Images/IkB-a/001-merger.tif]

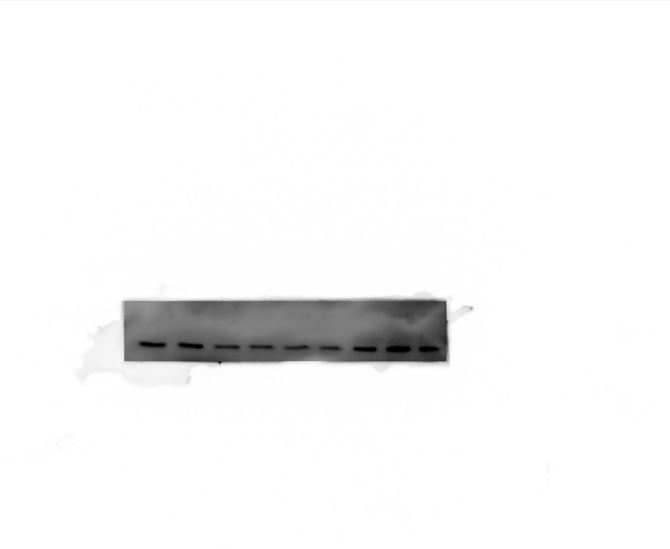

Supplement: Supplementary file 1 [file animals-12-02958-s001.zip › Western blot Original Images/IkB-a/001-shine.tif]

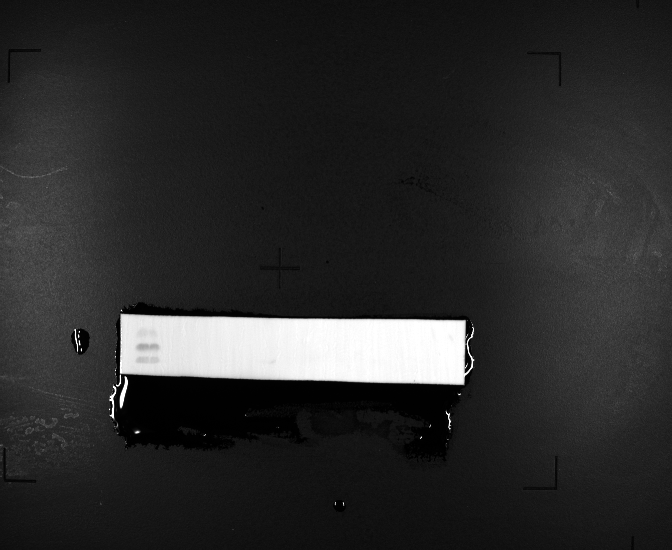

Supplement: Supplementary file 1 [file animals-12-02958-s001.zip › Western blot Original Images/P-AKT/001-bright.tif]

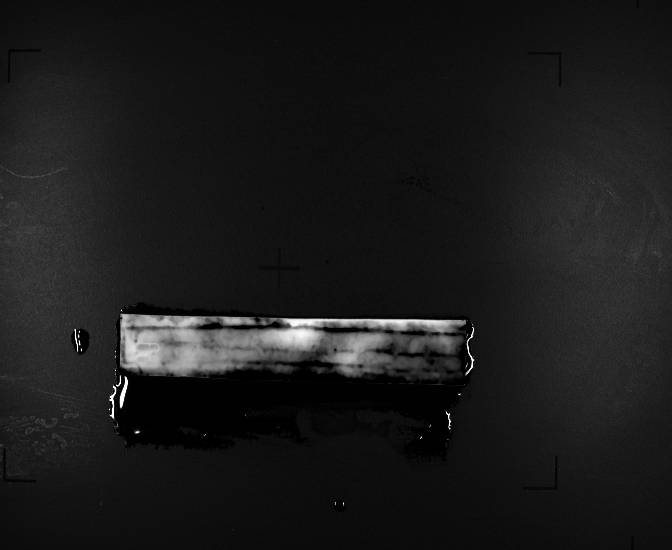

Supplement: Supplementary file 1 [file animals-12-02958-s001.zip › Western blot Original Images/P-AKT/001-merger.tif]

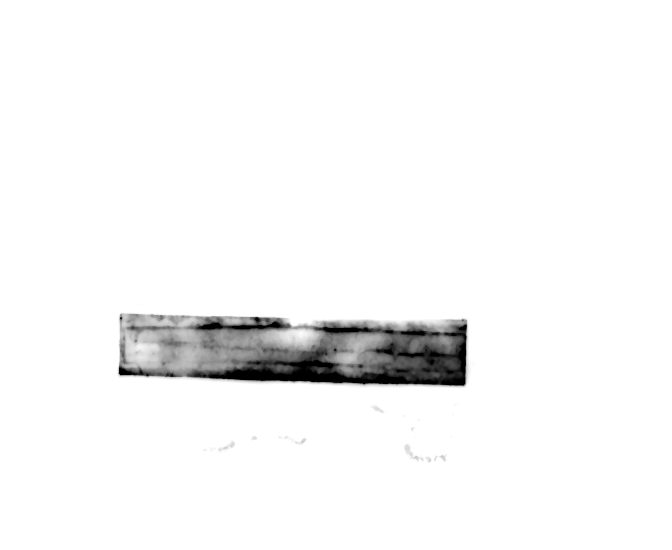

Supplement: Supplementary file 1 [file animals-12-02958-s001.zip › Western blot Original Images/P-AKT/001-shine.tif]

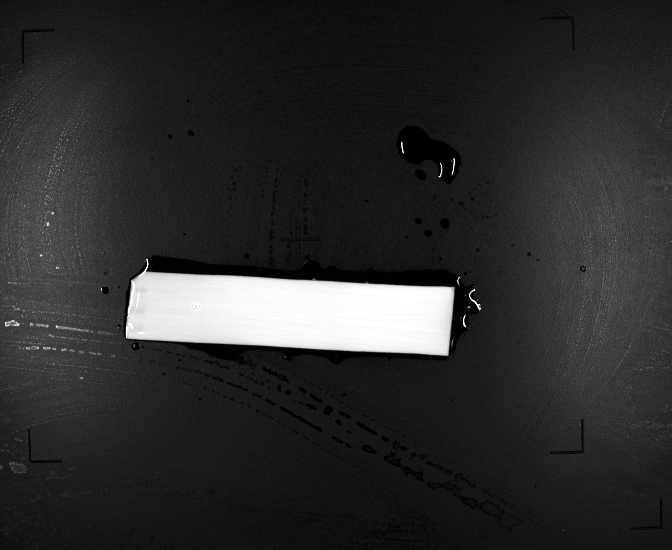

Supplement: Supplementary file 1 [file animals-12-02958-s001.zip › Western blot Original Images/P-IkB-a/001-bright.tif]

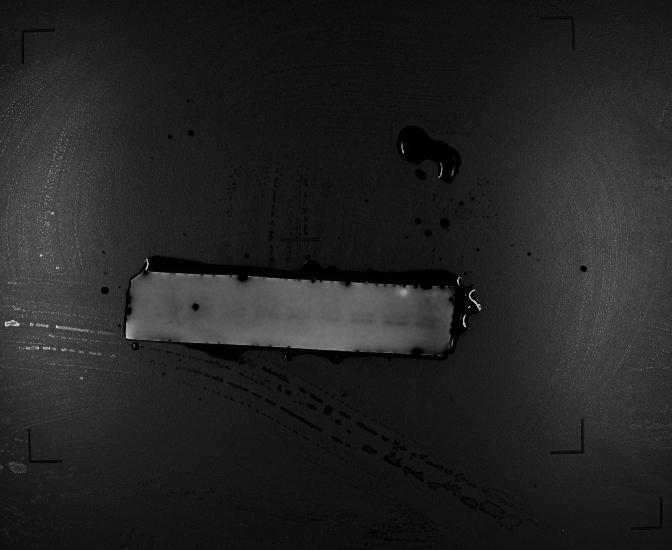

Supplement: Supplementary file 1 [file animals-12-02958-s001.zip › Western blot Original Images/P-IkB-a/001-merger.tif]

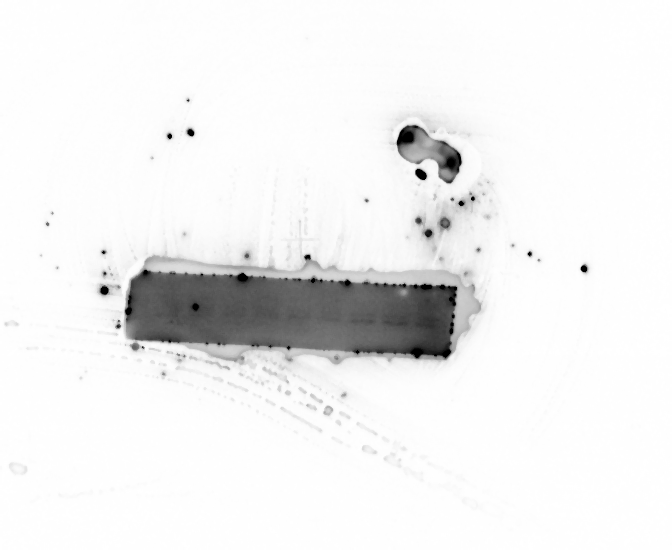

Supplement: Supplementary file 1 [file animals-12-02958-s001.zip › Western blot Original Images/P-IkB-a/001-shine.tif]

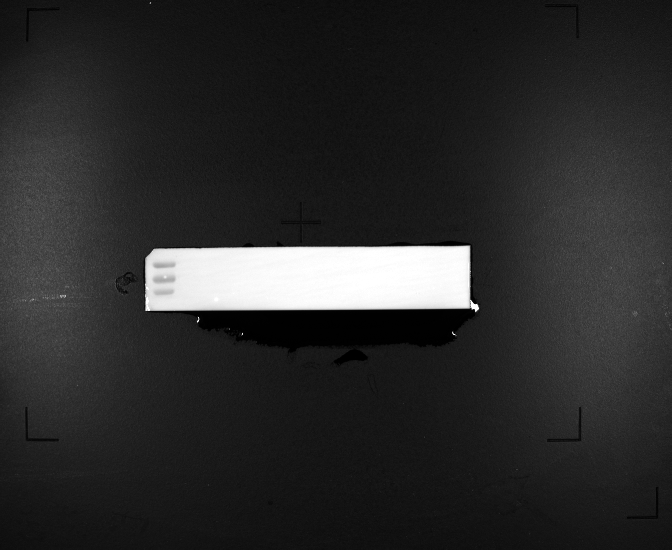

Supplement: Supplementary file 1 [file animals-12-02958-s001.zip › Western blot Original Images/P-Syk/001-bright.tif]

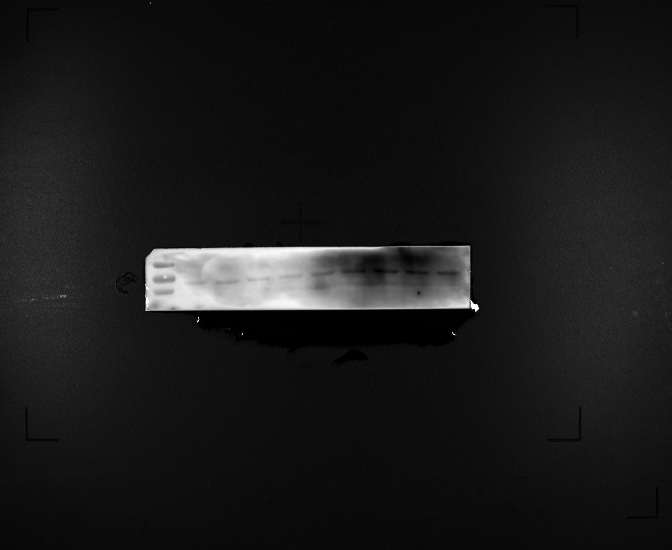

Supplement: Supplementary file 1 [file animals-12-02958-s001.zip › Western blot Original Images/P-Syk/001-merger.tif]

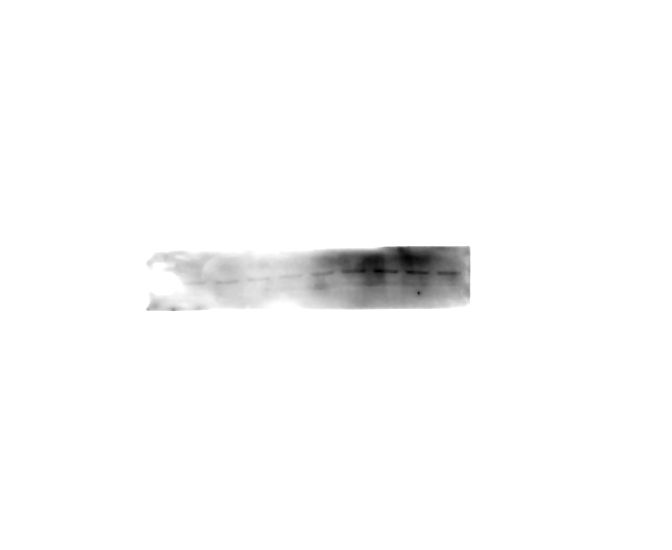

Supplement: Supplementary file 1 [file animals-12-02958-s001.zip › Western blot Original Images/P-Syk/001-shine.tif]

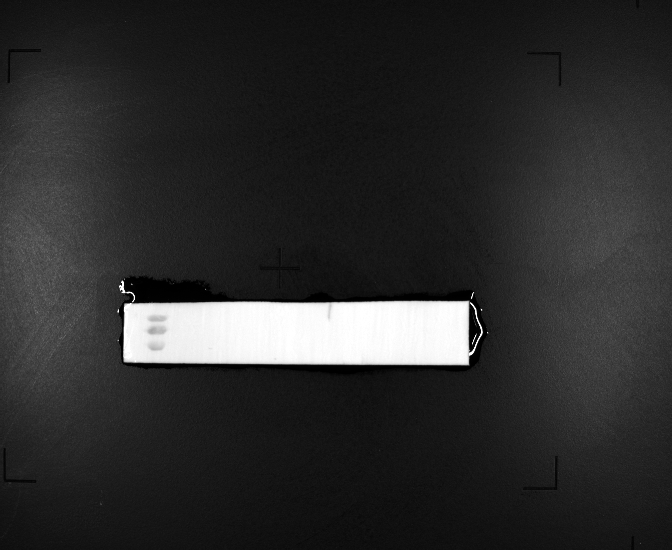

Supplement: Supplementary file 1 [file animals-12-02958-s001.zip › Western blot Original Images/Syk/001-bright.tif]

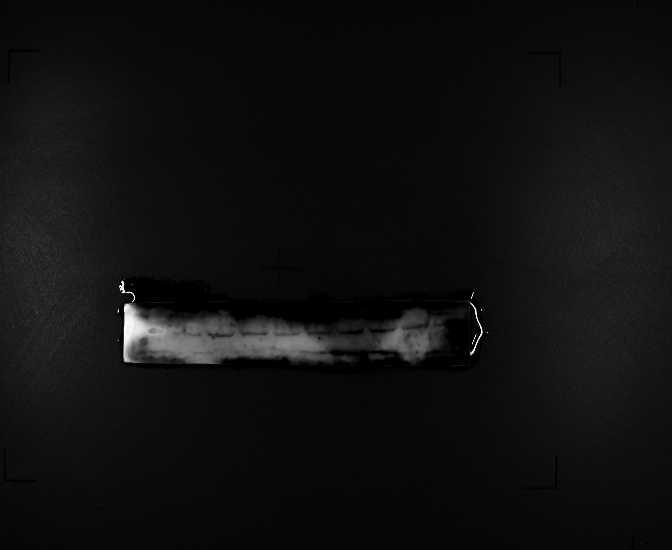

Supplement: Supplementary file 1 [file animals-12-02958-s001.zip › Western blot Original Images/Syk/001-merger.tif]

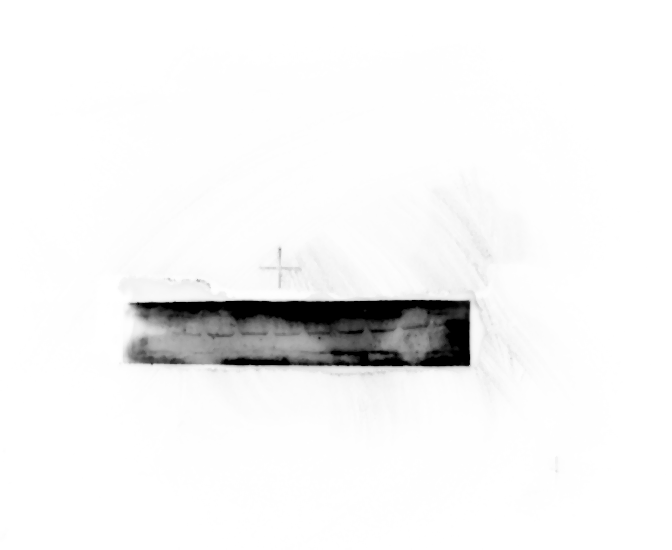

Supplement: Supplementary file 1 [file animals-12-02958-s001.zip › Western blot Original Images/Syk/001-shine.tif]
